# Post-colonoscopy colorectal cancers in a national fecal immunochemical test-based colorectal cancer screening program

**DOI:** 10.1055/a-2230-5563

**Published:** 2024-02-06

**Authors:** Pieter H. A. Wisse, Sybrand Y. de Boer, Marco Oudkerk Pool, Jochim S Terhaar sive Droste, Claudia Verveer, Gerrit A. Meijer, Evelien Dekker, Manon C. W. Spaander

**Affiliations:** 1 6993 Gastroenterology and Hepatology, Erasmus MC, Rotterdam, Netherlands; 2 Gastroenterology and Hepatology, Bevolkingsonderzoek Nederland, Rotterdam, Netherlands; 3 1228 Pathology, Netherlands Cancer Institute, Amsterdam, Netherlands; 4 26066 Gastroenterology and Hepatology, Amsterdam UMC Location AMC, Amsterdam, Netherlands

## Abstract

**Background** Post-colonoscopy colorectal cancers (PCCRCs) decrease the effect of colorectal cancer (CRC) screening programs. To enable PCCRC incidence reduction in the long-term, we classified PCCRCs diagnosed after colonoscopies performed in a fecal immunochemical test (FIT)-based screening program.

**Methods** PCCRCs diagnosed after colonoscopies performed between 2014–2016 for a positive FIT in the Dutch CRC screening program were included. PCCRCs were categorized according to the World Endoscopy Organization consensus statement into (a) interval PCCRC (diagnosed before the recommended surveillance); (b) non-interval type A (diagnosed at the recommended surveillance interval); (c) non-interval type B (diagnosed after the recommended surveillance interval); or (d) non-interval type C (diagnosed after the intended recommended surveillance interval, with surveillance not implemented owing to co-morbidity). The most probable etiology was determined by root-cause analysis. Tumor stage distributions were compared between categories.

**Results** 116362 colonoscopies were performed after a positive FIT with 9978 screen-detected CRCs. During follow-up, 432 PCCRCs were diagnosed. The 3-year PCCRC rate was 2.7%. PCCRCs were categorized as interval (53.5%), non-interval type A (14.6%), non-interval type B (30.6%), and non-interval type C (1.4%). The most common etiology for interval PCCRCs was possible missed lesion with adequate examination (73.6%); they were more often diagnosed at an advanced stage (stage III/IV; 53.2%) compared with non-interval type A (15.9%; *P* <0.001) and non-interval type B (40.9%; *P* =0.03) PCCRCs.

**Conclusions** The 3-year PCCRC rate was low in this FIT-based CRC screening program. Approximately half of PCCRCs were interval PCCRCs. These were mostly caused by missed lesions and were diagnosed at a more advanced stage. This emphasizes the importance of high quality colonoscopy with optimal polyp detection.

## Abstract (de)

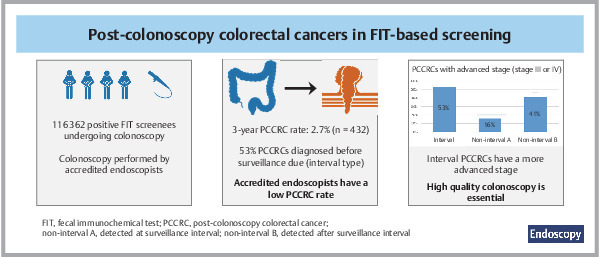

## Introduction

Over the past few decades, colorectal cancer (CRC) screening has been implemented in many countries worldwide 1 . Screening aims to reduce CRC incidence and CRC-related mortality by early detection and removal of colorectal polyps 2 . Many European countries have implemented fecal immunochemical test (FIT)-based screening programs, which offer colonoscopy to participants with a positive FIT result 1 . In screening programs, high quality performance of colonoscopies is essential to prevent the development of post-colonoscopy colorectal cancers (PCCRCs).

Classification of PCCRCs is important in establishing which quality measurements should potentially be adapted to enable a reduction in the number of PCCRCs. The World Endoscopy Organization (WEO) published a consensus statement to standardize terminology, identification, classification, and reporting of PCCRCs 3 . PCCRCs can be classified with respect to the surveillance interval to determine whether these intervals are appropriate. In addition, PCCRCs can be classified based on their most likely etiology to determine if endoscopists should improve their performance on certain quality indicators. For example, PCCRCs that develop in the right side of the colon after incomplete colonoscopies emphasize the importance of achieving a high cecal intubation rate 4 . PCCRCs that develop after procedures with polyp removal indicate the importance of complete polyp resection and timely follow-up after these resections 4 . PCCRCs that develop after missed lesions are related to polyp detection by endoscopists as reflected in their adenoma detection rate (ADR) and proximal serrated polyp detection rate 5 6 .

Studies reviewing PCCRCs are often based on small numbers and include a heterogeneous population undergoing colonoscopy 4 7 8 . In addition, it is important to include recent colonoscopies as PCCRC rates have declined over time 8 , probably owing to improvements in colonoscopy performance, accompanied by increased quality assurance and monitoring of endoscopists 9 10 . The aim of this study was to apply the WEO methodology to review PCCRCs diagnosed after colonoscopies performed after a positive FIT in a large nationwide CRC screening program.

## Methods

### Clinical setting

This population-based cohort study identified and reviewed PCCRCs diagnosed after a colonoscopy performed between 1 January 2014 and 31 December 2016 for a positive FIT result in the Dutch CRC screening program. This screening program started in 2014 and was gradually implemented to reach full roll-out in 2019 ( **Table 1s** , see online-only Supplementary material). Individuals aged 55–75 years receive a single FIT (FOB-Gold; Sentinel) biennially and a colonoscopy is offered to all participants with a positive FIT result. The cutoff for a positive result was 15 µg hemoglobin/g feces in the first 6 months of 2014 and was increased to 47 µg hemoglobin/g feces thereafter 11 .

Colonoscopies are performed by accredited endoscopists who are audited yearly. At these audits, the performance of individual endoscopists is discussed in relation to a set of colonoscopy quality indicators with minimum standards ( **Table 2s** ) 9 . During colonoscopy, detected polyps are removed and sent for pathologic evaluation. Treatment and surveillance are provided based on guidelines 12 . Colonoscopy and pathology data are centrally stored in a national screening database (ScreenIT; Topicus, Deventer, The Netherlands) 13 .

### PCCRC definition and identification

A PCCRC was defined as a CRC diagnosed during the study period and at least 6 months after an index colonoscopy (the first colonoscopy after the positive FIT result) without CRC diagnosis 3 . Appendicular cancers, neuroendocrine tumors, and anal cancers were excluded. PCCRCs were identified in the Dutch cancer registry up to 1 January 2020. Information regarding PCCRC location and stage, according to the 8th edition of the American Joint Committee on Cancer classification, was retrieved.

To classify PCCRC type, we used the clinical data collected during the yearly audits. At these audits, PCCRCs are provided to the endoscopist that performed the colonoscopy and discussed. The clinical data provided by the endoscopist enabled us to classify each PCCRC case.

### Clinical information

For all index colonoscopies, data were retrieved from the national screening database system (ScreenIT), which contains the endoscopist, completeness of procedure, bowel preparation, most advanced lesion diagnosed, and follow-up plan. Only the index colonoscopy was registered in the database. The most advanced lesion was CRC, followed by advanced adenoma, nonadvanced adenoma, and serrated polyp. Advanced adenomas had a size ≥10 mm and/or at least 25% villous histology and/or high grade dysplasia. A serrated polyp was a hyperplastic polyp, a sessile serrated polyp–adenoma, or a traditional serrated polyp. The right-sided colon was defined as the cecum, ascending colon, hepatic flexure, transverse colon, and splenic flexure.

For participants who developed a PCCRC subsequently, detailed information of each lesion described in the endoscopy and pathology report of this index colonoscopy was retrieved. Second screening or surveillance colonoscopies are not included in the national screening database. Therefor the clinical records of each participant who developed a PCCRC were reviewed at the healthcare service by the treating doctor to obtain the information needed to define the type of PCCRC.

### Surveillance interval

Surveillance intervals were based on the Dutch guidelines 6 . Incomplete colonoscopies were procedures where a large lesion was detected that could not be resected at the index colonoscopy or procedures without cecal intubation or with an insufficiently cleaned bowel (Boston Bowel Preparation Scale <6). These procedures resulted in a second colonoscopy within 6 months. Colonoscopies with resection of a large lesion (≥20mm in size) or piecemeal resection resulted in a surveillance interval of 12 months. For all other procedures, the recommended surveillance interval was assessed on the basis of the polyp score, which consisted of the number of adenomas, the polyp size (≥10mm), and the adenoma location and histology ( **Table 3s** ). This score was transformed into a recommendation for surveillance as follows: after 3 years (total score 3–5 points), after 5 years (total score 1–2), or FIT-based surveillance after 10 years (total score 0).

Colonoscopy surveillance was only offered if the expected balance between prevention of CRC versus risk of complications was positive, as decided by the treating physician. FIT-based surveillance was offered only to participants up to a maximum age of 75 years.

### Interval or non-interval PCCRC classification

Based on surveillance intervals, PCCRCs were categorized as interval or non-interval. Interval PCCRC was defined as a PCCRC diagnosed after an index colonoscopy in which no CRC was detected and before the date of the recommended surveillance/screening interval, as determined at the index colonoscopy. All other PCCRCs were non-interval PCCRCs. These were subdivided into: type A, diagnosed at the recommended surveillance interval; type B, diagnosed after the recommended surveillance interval; and type C, diagnosed after the intended recommended surveillance interval, with surveillance not implemented owing to patient co-morbidity. PCCRCs diagnosed before or around the appropriate surveillance interval in patients who did not undergo surveillance owing to co-morbidity were categorized as interval or non-interval type A.

For non-interval type A PCCRCs, a margin of 2 months was used for a surveillance interval of 12 months, or 6 months for surveillance intervals of 3 or 5 years. If an endoscopist advised a surveillance interval that was longer than recommended by the guideline, the surveillance interval as stated by the endoscopist was used to categorize the type of PCCRC. If an endoscopist advised a surveillance interval that was shorter than recommended by the guideline, the PCCRC diagnosed at this too early surveillance procedure was categorized as an interval type. All PCCRCs diagnosed 6 months after an incomplete procedure were categorized as non-interval type B as the guideline advised a second colonoscopy within 6 months.

### Root-cause analysis

An etiologic analysis was undertaken for each PCCRC, with four steps used to classify each PCCRC ( **Table 4s** ) 3 . Advanced adenoma was defined, according to the consensus statement, as an adenoma sized ≥10 mm and/or at least 25% villous histology and/or high grade dysplasia. An adequate colonoscopy was defined as a procedure with cecal intubation and with a sufficiently cleaned bowel (Boston Bowel Preparation Scale ≥6).

### PCCRC rate

In accordance with the WEO consensus statement, the PCCRC rate was determined by dividing the number of PCCRCs that occurred in the first 3 years after the index colonoscopy by the total of screen-detected CRCs and PCCRCs diagnosed in the first 3 years after the index colonoscopy 3 .

### Statistical analysis

Descriptive statistics were used to describe the results. The tumor stage of each PCCRC was determined according to category ([non]-interval categories and etiologic categories). Subsequently, the tumor stages of PCCRCs in the non-interval PCCRC group were compared with the tumor stages in the interval PCCRC group. The same was done for the etiologic categories. Chi-squared tests with a two-sided *P* value were used to compare the groups and *P* values <0.05 were considered to be statistically significant. Statistical analysis were performed in SPSS version 28.0.1.0.

### Ethics statement

The study was approved by the National Institute for Public Health and Environment on 18 April 2019 (number W1906_054).

## Results

**Fig. 1:**
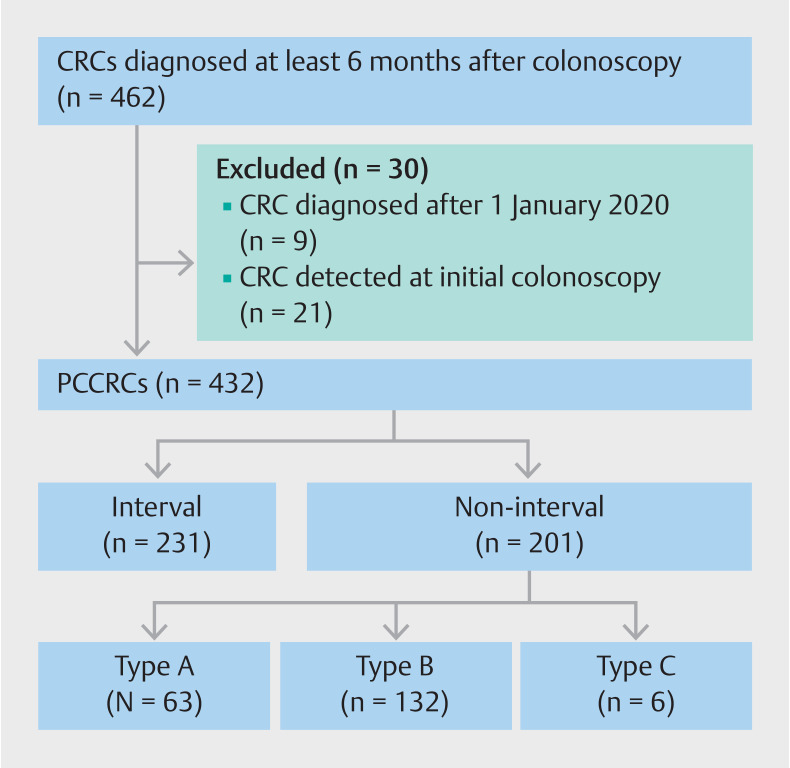
Flowchart of post-colonoscopy colorectal cancer (PCCRC) identification and subcategory classification. Interval PCCRC was defined as a PCCRC diagnosed before the surveillance interval as determined at the index colonoscopy. Non-interval type A was defined as a PCCRC diagnosed at the recommended surveillance interval. Non-interval type B was defined as a PCCRC diagnosed after the recommended surveillance interval. Non-interval type C was defined as a PCCRC diagnosed after the intended recommended surveillance interval, but with surveillance not implemented owing to patient co-morbidity.

Endoscopists performed 116362 index colonoscopies in FIT-positive screenees in the Dutch CRC screening program between 1 January 2014 and 31 December 2016. CRC was diagnosed in 9978 colonoscopies (8.6%). The remaining 106384 participants were followed for a median period of 51 months (interquartile range [IQR] 44–59 months). After linking with the Dutch Cancer Registry, 462 potential PCCRCs were identified. Of these potential PCCRCs, 21 were excluded as the cancer had already been diagnosed at the index colonoscopy and nine were excluded as the PCCRC was diagnosed after 1 January 2020, resulting in 432 PCCRCs ( Fig. 1 ). Of these, 279 were diagnosed within 3 years, resulting in a 3-year PCCRC rate of 2.7% (95%CI 2.4%–3.0%).

### PCCRC

The median time to PCCRC diagnosis was 31 months (range 6–67 months). Patients diagnosed with PCCRC had a median age at index colonoscopy of 67 years and 60% were men ( Table 1 ). The median hemoglobin level was 135 µg/g stool. In 44.9% of screenees with a PCCRC, an advanced adenoma was detected at the index colonoscopy. The majority (n=233; 53.9%) of PCCRCs were located in the right colon. Fewer PCCRCs were located in the left colon (n=99; 22.9%) and the rectum (n=89; 20.6%). For 11 PCCRCs (2.5%), the location was unknown ( Table 1 ).

**Table:** **Table 1** Characteristics of screening participants who underwent colonoscopy after a positive fecal immunochemical (FIT) result.

Characteristic	Colonoscopy (n=116362)	PCCRCs (n=432)
Age, median (IQR), years	67 (63–70)	67 (65–75)
Sex, male, n (%)	69943 (60.1)	260 (60.2)
FIT value, median (IQR), µg Hb/g feces	135 (70–201)	135 (75–201)
Most advanced finding at index colonoscopy, n (%)		
Colorectal cancer	9978 (8.6)	0 (0.0)
Advanced adenoma*	41982 (36.1)	194 (44.9)
Non-advanced adenoma	34552 (29.7)	124 (28.7)
Serrated lesion	3869 (3.3)	15 (3.5)
No lesion	18101 (15.6)	59 (13.7)
Unknown/other	7880 (6.8)	40 (9.3)
Time to PCCRC diagnosis, median (IQR), months		31 (19–39)
Location, n (%)†		
Right		233 (53.9)
Cecum		68 (15.7)
Ascending colon		89 (20.6)
Hepatic flexure		26 (6.0)
Transverse colon		33 (7.6)
Splenic flexure		17 (3.9)
Left		99 (22.9)
Descending colon		18 (4.2)
Sigmoid		71 (16.4)
Rectosigmoid		10 (2.3)
Rectum		89 (20.6)
Unknown		11 (2.5)
IQR, interquartile range; Hb, hemoglobin; PCCRC, post-colonoscopy colorectal cancer. * Advanced adenomas had a size ≥10mm and/or at least 25% villous histology and/or high grade dysplasia. † CRC location was only available for PCCRCs.		

### Interval or non-interval PCCRC classification

PCCRCs were categorized as interval PCCRCs (n=231; 53.5%) or non-interval PCCRCs (n=201; 46.5%). Non-interval type B (n=132; 30.6%) were more prevalent than non-interval type A (n=63; 14.6%) and non-interval type C (n=6; 1.4%) ( Fig. 1 ).

### Root-cause analysis

Six PCCRCs (1.4%) could not be categorized as the location of the previously detected advanced adenoma (n=1; 0.2%) or the PCCRC (n=5; 1.2%) was unknown. The etiology was most likely new CRC for 32 PCCRCs (7.4%). The remaining PCCRCs had as their most probable etiology: a possible missed lesion with adequate examination (n=202; 46.8%); a possible missed lesion with inadequate examination (n=28; 6.5%); a detected lesion that was not removed (n=57; 13.2%); and likely incomplete polyp resection (n=107; 24.8%) ( Table 2 ).

**Table:** **Table 2** Etiologic classification for interval and non-interval post-colonoscopy colorectal cancers (PCCRCs).

Etiologic classification	All PCCRCs (n=432)	Interval versus non-interval classification			
		Interval (n=231)	Non-interval PCCRCs (n=201)		
			Type A (n=63)	Type B (n=132)	Type C (n=6)
Possible missed lesion with adequate examination	202	170 (73.6)	22 (34.9)	9 (6.8)	1 (16.7)
Possible missed lesion with inadequate examination	28	0	0	28 (21.2)	0
Detected lesion that was not removed	57	0	0	57 (43.2)	0
Likely incomplete polyp resection	107	40 (17.3)	38 (60.3)	27 (20.5)	2 (33.3)
Likely new CRC	32	20 (8.7)	3 (4.8)	6 (4.5)	3 (50.0)
Unknown	6	1 (0.4)	0	5 (3.8)	0

The majority of interval PCCRCs (73.6%) had as their most probable etiology a possible missed lesion with adequate examination. Non-interval type A PCCRCs had as their most probable etiology a likely incomplete resection (60.3%); non-interval type B PCCRCs, a detected lesion that was not removed (43.2%) or a possible missed lesion with inadequate examination (21.2%). The majority of the non-interval type B PCCRCs (85/132; 64.4%), representing 20% of all PCCRCs, were therefore diagnosed in patients with an incomplete index colonoscopy, namely an index colonoscopy without cecal intubation or with insufficient bowel preparation (n=28), or with referral for surgery or endoscopic polypectomy (n=57). The 3-year PCCRC rate in our study would have decreased to 2.0% (95%CI 1.7%–2.3%) if these groups had received timely follow-up, within 6 months after incomplete colonoscopy.

### Stage distribution

The stage distribution for all PCCRCs was 33.3% stage I, 20.6% stage II, 28.7% stage III, 16.0% stage IV, and 1.4% unknown ( Table 3 ). Stage distribution differed between interval and non-interval PCCRCs. Interval PCCRCs (53.2%; reference) were significantly more often diagnosed at an advanced stage (stage III and IV) compared with non-interval type A PCCRCs (15.9%; *P* <0.001) and non-interval type B PCCRCs (40.9%; *P* =0.03).

**Table:** **Table 3** Stage distribution of interval and non-interval post-colonoscopy colorectal cancers (PCCRCs).

Stage	All PCCRCs (n=432)	Interval versus non-interval classification			
		Interval PCCRCs (n=231)	Non-interval PCCRCs (n=201)		
			Type A (n=63)	Type B (n=132)	Type C (n=6)
I	144 (33.3)	49 (21.2)	44 (69.8)	51 (38.6)	–
II	89 (20.6)	58 (25.1)	9 (14.3)	22 (16.7)	–
III	124 (28.7)	80 (34.6)	7 (11.1)	35 (26.5)	2 (33.3)
IV	69 (16.0)	43 (18.6)	3 (4.8)	19 (14.4)	4 (66.7)
Unknown	6 (1.4)	1 (0.4)	–	5 (3.8)	–

Advanced stage, stage III or IV, was less common in PCCRCs with as their most probable etiologies likely incomplete polyp resection (34.6%; reference) or a detected lesion that was not removed (35.1%; *P* =0.95) ( Table 4 ). Advanced stage was more common in PCCRCs with as their most probable etiologies a possible missed lesion with adequate examination (48.5%; *P* =0.02), a likely new CRC (62.5%; *P* =0.005), and a possible missed lesion with inadequate examination (60.7%; *P* =0.01).

**Table:** **Table 4** Stage distribution of post-colonoscopy colorectal cancers (PCCRCs) in the different etiologic classifications.

Stage	All PCCRCs (n=432)	Etiologic classification					
		Possible missed lesion with adequate examination (n=202)	Possible missed lesion with inadequate examination (n=28)	Detected lesion that was not removed (n=57)	Likely incomplete polyp resection (n=107)	Likely new CRC (n=32)	Unknown (n=6)
I	144 (33.3)	56 (27.7)	7 (25.0)	25 (43.9)	48 (44.9)	5 (15.6)	3 (50.0)
II	89 (20.6)	47 (23.3)	3 (10.7)	10 (17.5)	21 (19.6)	7 (21.9)	1 (16.7)
III	124 (28.7)	61 (30.2)	9 (32.1)	15 (26.3)	24 (22.4)	15 (46.9)	0
IV	69 (16.0)	37 (18.3)	8 (28.6)	5 (8.8)	13 (12.1)	5 (15.6)	1 (16.7)
Unknown	6 (1.4)	1 (0.5)	1 (3.6)	2 (3.5)	1 (0.9)	0	1 (16.7)

### Follow-up endoscopy

The recommended time to the next procedure, based on the guideline, in patients who developed a PCCRC was less than 6 months in 100 patients (23.1%), 12 months in 46 patients (10.6%), 3 years in 94 patients (21.8%), 5 years in 94 patients (21.8%), and 10 years in 98 patients (22.7%) ( Table 5 ; **Table 2s** ). The median time to diagnosis was 31 months (IQR 19–39) for all PCCRCs, 31 months (IQR 24–40) for interval PCCRCs, 37 months (IQR 13–39) for non-interval type A, 23 months (IQR 11–37) for non-interval type B, and 47 months (IQR 38–56) for non-interval type C.

**Table:** **Table 5** Recommended surveillance interval, according to the guideline, of interval and non-interval post-colonoscopy colorectal cancers (PCCRCs).

Surveillance interval*	All PCCRCs (n=432)	Interval versus non-interval classification			
		Interval PCCRCs (n=231)	Non-interval PCCRCs (n=201)		
			Type A (n=63)	Type B (n=132)	Type C (n=6)
<6 months	100 (23.1)	0 (0.0)	0 (0.0)	99 (75.0)	1 (16.7)
12 months	46 (10.6)	3 (1.3)	17 (27.0)	25 (18.9)	1 (16.7)
3 years	94 (21.8)	41 (17.7)	43 (68.3)	6 (4.5)	4 (66.7)
5 years	94 (21.8)	89 (38.5)	3 (4.8)	2 (1.5)	0 (0.0)
10 years	98 (22.7)	98 (42.4)	0 (0.0)	0 (0.0)	0 (0.0)
*A second colonoscopy within 6 months was offered after an incomplete colonoscopy: procedures with detection of a large lesion that could not be resected at the index colonoscopy; procedures without cecal intubation or with an insufficiently cleaned bowel (Boston Bowel Preparation Scale <6). A surveillance colonoscopy was offered after: 12 months following resection of a large lesion (≥20 mm) or a piecemeal resection; 3 years for participants with an adenoma score of 3–5 points; 5 years for those with a score of 1–2. FIT-based surveillance after 10 years was offered for participants with an adenoma score of 0. The adenoma score is shown in **Table 3s** .					

Interval PCCRCs were diagnosed in patients with a recommended surveillance interval of 12 months (n=3; 1.3%), 3 years (n=41; 17.7%), 5 years (n=89; 38.5%), and 10 years (n=98; 42.4%). Non-interval type A PCCRCs were diagnosed after 12 months (n=17; 27.0%), 3 years (n=43; 68.3%), and 5 years (n=3; 4.8%). Non-interval type B PCCRCs were diagnosed in patients with an indication for a second colonoscopy within 6 months (n=99; 75.0%) or with a recommended surveillance interval of 12 months (n=25; 18.9%), 3 years (n=6; 4.5%), or 5 years (n=2; 1.5%).

## Discussion

Identification and review of PCCRCs is strongly advised by the WEO to find opportunities to improve colonoscopy quality and prevent PCCRCs 3 . We identified 432 PCCRCs, in relation to 9978 screen-detected CRCs, in a screening population of over 110000 screenees undergoing colonoscopy in an organized nationwide FIT-based CRC screening program. The 3-year PCCRC rate was 2.7% and approximately half of the PCCRCs were interval PCCRCs, which were often caused by missed lesions after adequate colonoscopies and were diagnosed at a more advanced stage than non-interval type A and non-interval type B PCCRCs.

Previously reported 3-year PCCRC rates have varied from 7% to 9% in studies from Belgium, Denmark, Hong Kong, Sweden, and England 8 14 15 16 17 . The 3-year PCCRC rate in this study was 2.7%. This rate is considerably lower and falls below the minimum standard of 5.5% and the aspirational target of 3.6%, as suggested by Burr and colleagues 8 . Several factors may explain this low rate. First, endoscopists in the Dutch CRC screening program are accredited and monitored, which selects a group of high performing endoscopists 9 , which is reflected by high quality indicator performance by endoscopists in this program 6 . The ADRs of all endoscopists were above 40%, with a median of 67%, and a higher ADR has been associated with a lower risk of interval PCCRCs 6 . Second, we included only colonoscopies that were performed after a positive FIT for CRC screening, while the comparative studies used a range of colonoscopy indications. It is known that the PCCRC risk is higher after procedures performed for other indications, especially for patients with inflammatory bowel disease, where 3-year PCCRC rates of 25%–35% have been reported 8 18 .

The 3-year PCCRC rate was 3.6% in the English CRC screening program, which used a guaiac-based fecal occult blood test 8 . Colonoscopies in this program are also performed by accredited endoscopists 19 . The observed rate of 2.7% in our study is lower than the 3.6% observed in the English bowel cancer screening program. An explanation for this difference could be the timeframe in which the colonoscopies were performed as, in the English screening program, the 3.6% rate related to colonoscopies performed between 2005 and 2013. During this time period, important steps were made in the assessment of quality indicators, along with improvements in the colonoscopy procedure and technology 20 21 22 . The low PCCRC rates of the English and Dutch CRC screening programs underline the need for accreditation of endoscopists involved in CRC screening 17 . These models may serve as an example for quality assurance systems for other CRC screening programs 9 19 .

PCCRCs were not related to screenee factors such as age, sex, and FIT hemoglobin concentration. A German population that underwent screening colonoscopy with 10 years of follow-up showed an increased PCCRC risk for those at higher age and for women 23 . Over time, it will become clear whether these factor are also related to PCCRC risk in the Dutch FIT-based screening program. In Asian FIT-based screening populations, it has been shown that increased PCCRC risk was associated with men, the elderly, and screenees with higher FIT values 24 25 ; however, most procedures in these populations were performed in settings with low ADRs, so it could also be that screenee characteristics are less important when high quality colonoscopy is assured.

The majority of the PCCRCs (53.8%) were located in the right side of the colon, compared with around 25% of screen-detected cancers 26 . This is in line with previous studies and emphasizes the importance of cecal intubation and appropriate detection and removal of serrated polyps 5 25 27 28 . Advanced serrated polyps were not included in the etiologic classification as stated by the WEO, so the most likely etiology of PCCRCs that developed after advanced serrated polyp removal was a possible missed lesion in this study 3 . If these have been included, the etiologic classification of 15 PCCRCs would have changed from a possible missed lesion with adequate examination to a likely incomplete polyp resection. We propose that the etiologic classification in the WEO consensus statement should include these advanced polyps to ensure that PCCRCs that develop after removal of such polyps are classified as likely incomplete polyp resection, particularly as it is known that resection of these polyps is challenging and often incomplete 29 .

Most PCCRCs were interval PCCRCs (over 50%), followed by non-interval type B PCCRCs (approximately 30%) and non-interval type A (approximately 15%). Non-interval type C PCCRCs were scarce and represented only 1% of all PCCRCs. This distribution is largely similar to that observed in the Basque FIT-based CRC screening program 30 .

Approximately 45% of PCCRCs were diagnosed at stage III or IV compared with over 50% of clinically diagnosed CRCs and approximately 30% of screen-detected CRCs 26 . This high proportion among PCCRCs contrasted with a study from Korea, which showed a more favorable stage distribution in PCCRCs compared with screen-detected cancers 25 . Unfortunately, this study did not perform a root-cause analysis, which would enable a more detailed comparison; however, the Korean surveillance guideline is less restrictive than the Dutch guideline, so surveillance may have been performed earlier and resulted in more earlier stage non-interval type A PCCRCs 31 .

In our study, the proportion of cancers detected at an advanced stage was significantly higher for interval PCCRCs compared with non-interval type B and, especially, non-interval type A PCCRCs. The majority of these interval PCCRCs, 74%, had as their most probable etiology a missed lesion with adequate examination. It therefore seems that increased detection of precursor lesions, reflected in the ADR and proximal serrated polyp detection rate, is needed to reduce interval PCCRCs 5 6 . Improvements in this direction should be optimization of endoscopists’ colonoscopy technique. In addition, the use of high definition colonoscopes and artificial intelligence will contribute to increased detection of precursor lesions 32 33 .

Almost 50% of PCCRCs in our study had as their most probably etiology a possible missed lesion with adequate examination. This is in line with several other studies that reported this etiology as being the most prevalent 34 35 36 37 . The proportion of PCCRCs with an incomplete polyp resection as their most probable etiology was 25% in our study, which was higher than reported in previous studies 4 34 35 36 . This could be explained by the high proportion of participants in whom advanced adenomas were detected at the index colonoscopy, almost 40%, in this FIT-positive population, and demands improvement in polyp resection techniques 29 .

Approximately 20% of PCCRCs (n=99) were diagnosed after incomplete index colonoscopies. These cancers represent a large proportion (75%) of the non-interval type B PCCRCs in this study. This emphasizes the importance of timely scheduling and adherence to second colonoscopies for large polyp removal or complete inspection as this may prevent progression to (more advanced) CRC in these patients. The 3-year PCCRC rate in our study would have decreased to 2.0% if these groups had received a timely, within 6 months, second colonoscopy after the incomplete colonoscopy. Therefore, in the context of colonoscopies performed in FIT-based CRC screening, a potential benchmark for the 3-year PCCRC rate could be the observed rate of 2.7%, with an aspirational target of 2.0%.

The availability of a large comprehensive digital reporting system of colonoscopies performed in the context of CRC screening with linkage to the nationwide cancer registry provided us with the opportunity to perform this study. The detailed data can be used at both an endoscopist or service level for quality auditing and at an individual level for root-cause analysis. Besides that, we included a large homogeneous FIT-positive screening population, which enabled us to provide a precise estimate of the PCCRC rate in this population. Therefore, we are able to propose a minimum standard and aspirational target for the 3-year PCCRC rate in this setting. As colonoscopies were performed within only the last 5–10 years, the results presented resemble current clinical practice.

A limitation in the generalizability of our study could be the age distribution of participants. The proportion of participants aged 75 was high in the first years after the start of the Dutch screening program. A larger proportion of these participants that underwent colonoscopy may not have been eligible for surveillance colonoscopy owing to age and co-morbidity restrictions, which could have affected the observed 3-year PCCRC rate. In addition, we were not able to assess deviation from the surveillance guideline for all colonoscopies as second screening and surveillance colonoscopies were not included in the national screening database. We were however able to retrieve these data for the PCCRCs, which enabled us to accurately define interval versus non-interval PCCRCs and resulted in data that were not affected by deviation from the surveillance guideline by endoscopists. Finally, the distribution of PCCRCs into interval and non-interval groups may change when follow-up time increases; however, our follow-up, with a median of 51 months, ensures that colonoscopies resemble current endoscopist performance and was sufficient to determine a 3-year PCCRC rate and perform a root-cause analysis.

In conclusion, the 3-year PCCRC rate was only 2.7% for colonoscopies performed in a FIT-based CRC screening program, which is lower than has been reported in other studies. Accreditation and auditing seem important to reduce PCCRC risk. Approximately half of PCCRCs were interval and the majority of these had as their most likely etiology a possible missed lesion with adequate examination. These interval PCCRCs were diagnosed at a more advanced stage than non-interval type A and non-interval type B PCCRCs, which emphasizes the importance of high quality colonoscopy with optimal polyp detection.

## Supplementary Material

Supplementary material Zusatzmaterial
