# Supplementary material for: Post-colonoscopy colorectal cancers in a national fecal immunochemical test-based colorectal cancer screening program
Source: Endoscopy. 2024 Feb 6;56(5):364–72. doi: 10.1055/a-2230-5563 (PMC11583002; doi:10.1055/a-2230-5563)
Supplement: Supplementary file 1 — Supplementary material [file 10-1055-a-2230-5563_22460144.pdf_suppl.pdf.pdf]

Supplementary material

Post-colonoscopy colorectal cancers in a national FIT-based CRC screening program

Pieter H.A. Wisse, Sybrand Y. de Boer, Marco Oudkerk Pool, Jochim S. Terhaar sive Droste, Claudia Verveer, Gerrit A. Meijer, Evelien Dekker, Manon C.W. Spaander

Table 1s: Invited age cohorts and age of FIT-positive participants that underwent colonoscopy during the implementation of the Dutch colorectal cancer screening program.

	**2014**	**2015**	**2016**
**Age at invitation***			
First invitation	63, 65, 67, 75, 76	61, 63, 65, 67, 69, 75	59, 61, 63, 71, 75
Second invitation			65, 67, 69
**Age at colonoscopy**			
Mean	70.8	66.6	66.5
Median (IQR)	74.5 (65.8-75.7)	65.9 (63.7-68.9)	65.8 (61.7-70.7)

IQR = interquartile range

* Invitees were selected based on their birth cohort, so all citizens born in 1938, 1939, 1947, 1949 and 1951 were invited in 2014. Citizens aged 63, 65 or 67 years in 2014 (born in 1947, 1949 or 1951) were reinvited in 2016 (aged 65, 67 or 69).

Supplementary material

Table 2s: Overview of quality indicators and corresponding minimum standards for endoscopists performing colonoscopy in the Dutch CRC screening program.[1]

Quality indicator	Definition	Threshold
**Completeness of examination**		
Cecal intubation rate	Proportion of colonoscopies with cecal intubation	≥95%
Rate of sufficient bowel preparation	Proportion of colonoscopies in which the colon was sufficiently clean to inspect the mucosa (BBPS≥6)	≥90%
Rate of sufficient cecal withdrawal time	Proportion of colonoscopies with cecal intubation and without lesions with a withdrawal time ≥6 minutes	≥90%
**Detection rates**		
Cancer detection rate	Proportion of colonoscopies in which (at least) one cancer was detected	Monitoring
Adenoma detection rate	Proportion of colonoscopies in which (at least) one adenoma was detected	≥30%
Mean number of adenomas per procedure	Mean number of adenomas per colonoscopy	Monitoring
Mean number of adenomas per positive procedure	Mean number of adenomas per positive colonoscopy	Monitoring
**Removal rate**		
Polyp removal rate	Proportion of colonoscopies in which no second colonoscopy was planned to remove a polyp	≥90%
**Patient satisfaction**		
Comfort rate	Proportion of colonoscopies in which the patient experienced no to mild discomfort (GCS≤3)	Monitoring

BBPS = Boston bowel preparation scale; CRC = colorectal cancer; GCS = Gloucester comfort scale

Supplementary material

Table 3s: Scoring system based on polyp characteristics determined by colonoscopy and pathology evaluation

Polyp characteristics	Options	Corresponding Score****
**Number of adenomas**	0-1	0
	2-4	1
	≥5	2
**Presence of at least one adenoma or serrated polyp* ≥10mm**	No	0
	Yes	1
**Presence of at least one villous adenoma****	No	0
	Yes	1
**Presence of at least one proximal adenoma*****	No	0
	Yes	1

* Serrated polyps are defined as hyperplastic polyps, sessile serrated polyps/adenomas and traditional serrated polyps.

** Villous adenomas are defined as ≥75% villous histology.

*** Proximal is defined as located in the cecum, ascending colon, transverse colon or splenic flexure.

**** Based on the total number of points the related surveillance interval can be recommended. Surveillance after three years (total score 3-5 points), after five years (total score 1-2), or FIT-based surveillance after ten years (total score 0).

Supplementary material

Table 4s: Etiology classification of PCCRCs[2]

Step	Question	Answer	Proceed to / Classify as
1	Was the interval between index colonoscopy and PCCRC >4 years?	Yes	A likely new CRC
		No	Step 2
2	Was an advanced adenoma seen during the index colonoscopy in the segment (or adjacent segment) in which the PCCRC was diagnosed?	Yes	Step 4
		No	Step 3
3	Was the cecum intubated and bowel preparation good* at index colonoscopy?	Yes	Possible missed lesion with adequate examination
		No	Possible missed lesion with inadequate examination
4	Was the lesion resected?	Yes	Likely incomplete polyp resection
		No	Detected lesion that was not removed

* Defined as Boston bowel preparation scale ≥ 6.

References

1. Bronzwaer MES, Depla A, van Lelyveld N et al. Quality assurance of colonoscopy within the Dutch national colorectal cancer screening program. Gastrointest Endosc 2019; 89: 1-13. DOI: S0016-5107(18)33037-2 [pii]

2. Rutter MD, Beintaris I, Valori R et al. World Endoscopy Organization Consensus Statements on Post-Colonoscopy and Post-Imaging Colorectal Cancer. Gastroenterology 2018; 155: 909-925 e903. DOI: S0016-5085(18)34571-2 [pii]
